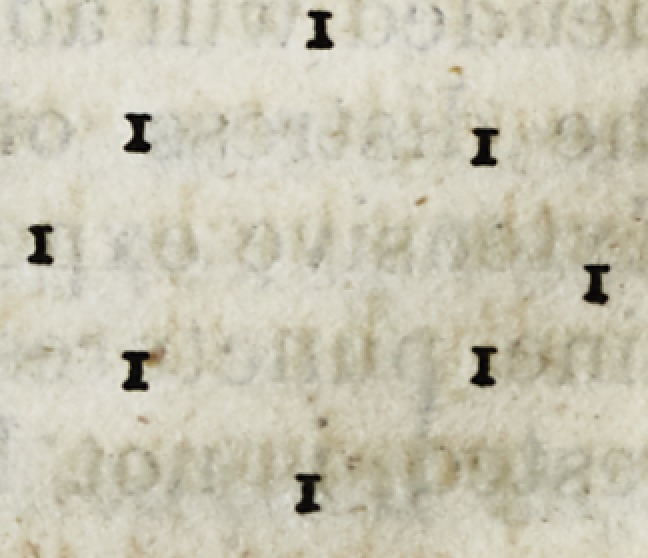# On the Practice of Vaccination

**Published:** 1826-11

**Authors:** George Gregory

**Affiliations:** Physician to the Small-Pox and Vaccination Hospital.


### VACCINATION.

*On the Practice of Vaccination.* By George Gregory, M.D. Physician to the Small-Pox and Vaccination Hospital.

In the performance of this very simple, but most important operation, it is at all times highly desirable, and in many cases an object of the first importance, to *ensure* its success. Failure *in the operation*, (for of that only I am here to speak,) is always harassing to the parent. Frequently it is taken as a pretext for deferring the process altogether; and, in the case of small-pox existing in the neighbourhood, it exposes the life of the child to great and imminent hazard. With this impression, I have, for several years past, directed my attention, in an especial manner, to the causes of failure in conducting the operation of vaccination; and I believe I have now detected the most of them. If there are any which have escaped my notice, the omission may perhaps hereafter be supplied through the kindness of some of the correspondents of this Journal. Of these sources of failure, some have reference to the mode of operating,—some to the selection of lymph,—and others to the system of the subject operated upon.

1. The first and most general cause of failure is, I should presume, the use of the dry lymph of points and glasses, especially of the former. Not having any experience, however, in this mode of operating, I am incapable of forming a judgment of the extent to which it applies. It would be highly satisfactory to myself, (and probably not less so to other readers,) if some country practitioner would be kind enough to state in what proportion the points and glasses, with which he may have been supplied from London, (especially by the National Vaccine Establishment,) are *effectual* in producing a vesicle; which of the two is to be preferred; and what is the most successful mode of operating with them.

I have ascertained, by frequent experiments, that lancets, well charged, and kept perfectly cool, will retain the virus unaltered, even in summer, for about six hours.

2. Most vaccinators, however, must have occasionally experienced failures, when operating in the *direct* mode; one of the most frequent causes of which is the employment of an unfit lancet. This is a matter of the utmost consequence to the success of the operation. Unless the lancet be clean, and moreover perfectly sharp, the virus is *thrown back* upon the shoulder of the instrument, and not a particle enters the wound. Some surgeons, to remedy this, smear the remaining virus over the *surface* of the skin operated upon; but frequent observation has proved to me the utter inutility of this measure. It is curious, and instructive also, to notice the different degrees of *toughness* in the skins of children. In a very large proportion of the cases of failure which have fallen under my own immediate observation, I have remarked an unusual toughness in the child's skin, and have consequently attributed the want of success to the use of a comparatively *blunt* lancet. It has frequently occurred to me to notice that a lancet, which has been successfully employed in venesection, is yet not sufficiently sharp for the purposes of vaccination. It is desirable that a vaccinating lancet should have a *broad* shoulder, for an instrument of this form best retains an adequate portion of virus.

3. Another source of failure may be found in the mode which some practitioners have of operating upon the unstretched skin. To ensure success, the skin should be kept, during the whole time, *perfectly tense*; and this is only to be done by grasping the arm firmly, and fixing the skin between the thumb and fore-finger of the left hand. In the hollow thus formed, there is ample room for as many insertions as may be desirable.

It does not coincide with the strict object of this paper, but it cannot be altogether irrelevant, to point out the proper number of incisions which should be made. A series of observations which I have made at the Small-Pox Hospital, during the last six months, has satisfied me that the most complete effect, both upon the arm and upon the constitution, is made by *six* or *eight* punctures, supposing them all to be effectual. In the space just alluded to, eight, or even nine, punctures may easily be made in the circular form, and at moderate distances; as thus—

The advantage of the circular form is, that the true figure of the areola is thus preserved: independent of which, all forms of cutaneous eruption are naturally disposed to assume either a circular or a crescentic arrangement.

On this subject I have only further to add, that it is a matter of perfect indifference, provided the directions here given are followed, whether little or much blood flows from the wounds.

4. Another class of circumstances which I have observed to interfere with the success of the operation, has reference to the *selection of lymph.* It is unnecessary for me to dwell at length on the impropriety of taking lymph from a vesicle, of which the areola has begun to subside. After the tenth day, the virus is scarcely fluid; and the moisture which sometimes exudes at this period cannot be relied on for propagating the disease. All practitioners are aware that the lymph ought to be perfectly limpid; and this cannot be ensured after the eighth day (including the day of insertion).

5. But, though this matter is well enough understood, I question whether practitioners have sufficiently considered another point,—viz. when a well-chosen vesicle ceases to afford effective lymph. The facts I believe to be these:—A fifth-day vesicle will not commonly afford virus for more than one subject. An eighth day vesicle (even when very tumid) cannot be relied upon for more than six or seven subjects. If the lancet is applied to it oftener than this, (especially with any degree of roughness,) an effusion of common serum takes place, and the lymph becomes too much diluted to produce any effect. It may not be altogether superfluous to remind the reader, that effective lymph must always possess a definite degree of intensity, and that the maximum of dilution is often rapidly attained. To prevent this too-frequent source of disappointment, the vesicle should at all times be handled very gently. It is obvious that, the younger the lymph (fourth or fifth day), the greater is its degree of *intensity*.

The propriety of inserting a numerous crop of punctures becomes here very evident. Besides saturating the system more effectually, it enables the vaccinator, at a large establishment, to open a new vesicle every third or fourth operation; the advantages of which, in ensuring the success of the process, are great and undeniable. It may perhaps be imagined by some, that the numerous punctures now recommended will add considerably to the local inflammation, and the distress of the child. This, however, is not the case. Extensive experience has proved to me, that the areola from nine punctures, if arranged in the manner I have just suggested, is not larger than that from two or three placed at a considerable distance from each other. The greatest number of effective insertions I have hitherto made is twenty, and I have noticed that, though the *constitution* sympathises more decidedly in such a case, the *local* irritation is not, *cæteris paribus,* greater here than under common circumstances. In a few of these cases, I have observed, about the eighth day, a pretty copious eruption all over the body, of a lichenous character, disposed in crescentic forms, and receding in two or three days. I am strongly inclined to view this as a peculiar disease—Vaccine Lichen, the constitutional effect of a copious production of the vaccine virus. The objection, however, that parents generally raise to these numerous punctures, has hitherto prevented me from determining this point in a manner that can be considered perfectly decisive.

6. The last source of failure in the operation of vaccination may be traced to the *system of the subject operated upon.* To ensure the success of the operation, the child should be in perfect health. So far from thinking it desirable (as many persons tell us, and as the common people in this town very generally believe,) to vaccinate during the presence of hooping cough, I am sure it is quite the reverse. Robust health is the best *predisposer* of successful vaccination. We all know that, when small-pox is once in the blood, vaccination will never prosper. The vesicles, if they rise, are then tardy and imperfect. In like manner, vaccination *sometimes* fails from the prior occupation of the system by some other internal disorder; and failure, therefore, does not *necessarily* presume error on the part of the practitioner. This, however, is a flattering unction, and far from being a common case; for the drooping of the little patient would generally be so obvious, as to discourage both parents and practitioners from attempting the operation.

If it had been my object in this paper to investigate the sources of failure in the protecting influence of vaccination, I should have taken this opportunity of making some remarks on the ages best adapted for obtaining the full effect of this salutary process; but I wish to confine myself entirely to the causes of failure in the operation itself. I shall merely remark, therefore, in reference to that question, that the most proper age for vaccination is between the second and fifth month; that is to say, after the infant has acquired plumpness, and before it has begun teething.

I may perhaps be permitted to conclude this communication with the following short "Instructions for young Vaccinators."

See that the child be in good health, and free from any cutaneous affection. Select from a healthy child, lymph of the sixth, seventh, or eighth day. Be careful that your lancet be extremely sharp, and if it be broad-shouldered, so much the better. Let there be a tangible *drop* at the point of the lancet, and be not satisfied with a mere moistening of the instrument. Let the skin be kept perfectly tense during the time of insertion, by grasping the arm of the child firmly, and extending the skin between the thumb and first finger of the left hand. Let the lancet be inserted from above downwards, and at each fresh insertion dip the point of the lancet in the lymph that remains around the incision *first* made. Make from six to ten punctures in a circular form, enclosing a space about the size of a shilling. At each insertion, press the point of the lancet firmly against the *lower* surface of the wound.

"Then the charm is firm and good."

If possible, open a fresh vesicle every second or third operation.

8, *Upper John-street, Golden square;*

*October* 10*th*, 1826.

## Figures and Tables

**Figure f1:**